# Supplementary material for: A Starch- and Sucrose-Reduced Diet Has Similar Efficiency as Low FODMAP in IBS—A Randomized Non-Inferiority Study
Source: Nutrients. 2024 Sep 9;16(17):3039. doi: 10.3390/nu16173039 (PMC11397485; doi:10.3390/nu16173039)
Supplement: Supplementary file 1 [file nutrients-16-03039-s001.zip › nutrients-3166947-supplementary.pdf]

Supplementary Table S1. Recommendations of fruit intake according to a starch- and sucrose-reduced diet

Well tolerated	Tolerated by some	Not tolerated
Avocado	Persimmons	Apples
Blackberries	Plums	Apricots
Blueberries	Raisins	Bananas
Boysenberries	Watermelon	Cantaloupe
Cherries		Dates
Cranberries		Grapefruit
Currants		Guava
Figs		Honeydew melon
Gooseberries		Mangos
Grapes		Nectarines
Kiwi		Oranges
Lemons		Peaches
Limes		Pineapple
Loganberries		Tangelos
Olives		Tangerines
Papaya		
Pears		
Pomegranates		
Prunes		
Raspberries		
Rhubarb		
Strawberries		

Supplementary Table S2. Recommendations of vegetable and legume intake according to a starch- and sucrose-reduced diet

**Well tolerated**	**Tolerated by some**	**Not tolerated**
Alfalfa sprouts	Edamame soybeans	Beets
Artichokes*	Jicamas	Black beans
Arugulas	Leeks	Black-eyed peas
Asparagus*	Okra	Butternut
Bamboo shoots	Pumpkin	Carrots
Bok choy	Snow peas	Cassava
Broccoli*	Tempeh	Chickpeas
Brussel sprouts*	Tofu	Corn
Cabbage*	Yellow wax beans	Garlic
Cauliflower*		Green peas
Celery		Lentils
Chard		Kidney beans
Chicories		Lima beans
Chives		Navy beans
Collard greens		Onion
Cress		Parsnips
Cucumber		Pinto beans
Eggplant		Potatoes
Endive		Soybeans
Green beans		Split peas
Kale		Sweet potatoes
Lettuce		Yams
Mung bean sprouts		
Mushrooms		
Mustard green		
Peppers		
Radishes		
Spaghetti squash		
Spinach		
Tomatoes		
Turnips		
Yellow squash		
Zucchini		

*excess intake can cause bloating/flatulence in all individuals

Supplementary Table S3. Previous or present comorbidity and drug treatments

**Comorbidity**	**IBS N=155**
Allergy	17 (11.0)
Anxiety	7 (4.5)
Asthma bronchialis	7 (4.5)
Burned out	10 (6.5)
Depression	10 (6.5)
Eczema	19 (12.3)
Fibromyalgia	9 (5.8)
Hypertension	12 (7.7)
Hypothyroid disease	6 (3.9)
Lactose intolerance	10 (6.5)
Migraine/headache	10 (6.5)
Reflux/hiatushernia	18 (11.6)
**Drug treatment**	
Allergy medicines	24 (15.5)
Antidepressants	23 (14.8)
Asthma inhalators	8 (5.2)
Hormonal treatment*	24 (15.5)
Laxatives/bulking agents	38 (24.5)
Levaxine	6 (3.9)
NSAID	50 (32.3)
Paracetamols	54 (34.8)
Proton pump inhibitors	48 (31.0)
**Dietary supplements**	
Iron	7 (4.5)
Other minerals (Ca, Mg, Z, Si)	26 (16.8)
Vitamin B/Folic acid	14 (9.0)
Vitamin D	26 (16.8)
Multivitamins	23 (14.8)
Vitamin D and/or multivitamins	39 (25.2)
Probiotics	15 (9.7)

IBS = irritable bowel syndrome. *= combination pills. Values are given as number (percentage).

Supplementary Table S4. Energy and nutrient intake

	SSRD N = 77			Low FODMAP N = 78			P-value*
**Variables**	**Values**	**P-value**	**Difference**		**P-value**	**Difference**	
**Energy (kcal)**							
Baseline	1813±535			1783±510			0.718
4 weeks	1534±478	<0.001	-284±549	1667±500	0.036	-128±491	0.082
6 months	1792±600	0.535	-56±608	1676±739	0.069	-192±705	0.323
**Carbohydrates (g)**							
Baseline	178±59			179±53			0.923
4 weeks	90±38	<0.001	-88±61	155±54	<0.001	-25±46	<0.001
6 months	157±63	0.044	-21±69	160±70	0.014	-26±70	0.736
**Protein (g)**							
Baseline	71(54-81)			66(52-80)			0.316
4 weeks	79(61-81)	0.002	7(-4-27)	68(51-81)	0.471	2(-11-16)	0.083
6 months	74(54-86)	0.573	-3(-15-14)	66(49-81)	0.604	-3±26	0.728
**Fat (g)**							
Baseline	76(60-93)			73(54-93)			0.656
4 weeks	83(67-109)	0.141	5(-12-34)	76(52-93)	0.308	-7(-22-20)	0.007
6 months	88(54-112)	0.922	-2(-22-23)	71(48-98)	0.071	-16(-27-19)	0.111
**Fiber (g)**							
Baseline	18(12-23)			18(15-22)			0.745
4 weeks	16(12-22)	0.162	-1(-6-3)	15(11-20)	0.001	-3(8-2)	0.162
6 months	18(13-24)	0.721	0 (-5-4)	15(10-20)	<0.001	-4(-7-2)	0.026
**Whole grain (g)**							
Baseline	25(13-44)			28(17-45)			0.309
4 weeks	22(8-39)	0.244	-6(-19-15)	30(14-54)	0.562	2(-13-17)	0.190
6 months	25(11-43)	0.538	1(-19-17)	25(13-44)	0.575	3(-25-18)	0.954
**Sucrose (g)**							
Baseline	25(14-40)			26(15-41)			0.405
4 weeks	6(3-9)	<0.001	-17 (-35-(-7))	18(12-36)	0.027	-5(-15-5)	<0.001
6 months	17(11-32)	0.011	-4 (-15-3)	22(10-36)	0.080	-6(-16-10)	0.881
**Starch (g)**							
Baseline	46±23			51±25			0.197
4 weeks	20±20	<0.001	-25±28	48±25	0.515	-3±32	<0.001
6 months	41±25	0.298	-5±33	42±28	0.008	-13±32	0.243
**Monosaccharides (g)**							
Baseline	27(18-38)			27(17-35)			0.523
4 weeks	17(10-34)	<0.001	-5(-14-2)	21(15-28)	0.001	-3(-12-2)	0.375
6 months	29(16-35)	0.249	-3(-12-6)	24(15-35)	0.216	-3(-9-4)	0.869
**Disaccharides (g)**							
Baseline	36(24-54)			41(28-58)			0.271
4 weeks	16(12-27)	<0.001	-17(-35-(-6)	31(23-49)	0.119	-2(-17-10)	<0.001
6 months	29(20-45)	0.017	-5(-18-3)	30(19-53)	0.107	-6(-21-12)	0.918
**Added Sugar (g)**							
Baseline	33(16-46)			32(17-52)			0.955
4 weeks	5(2-11)	<0.001	-24(-43-(-10)	24(13-43)	0.134	-2(-17-6)	<0.001
6 months	18(8-41)	0.023	-7(-21-8)	24(9-53)	0.472	-4(-17-14)	0.265
**Alcohol (g)**							
Baseline	4(0-16)			3(0-11)			0.551
4 weeks	0(0-10)	0.039	0(-6-0)	3(0-11)	0.760	0(-4-5)	0.139
6 months	3(0-16)	<0.001	0(-12-4)	5(0-14)	<0.001	2(-2-9)	0.103

SSRD = starch-and sucrose-reduced diet with 68 patients at week 4 and 46 at month 6. Low FODMAP = low content of fermentable oligo-, di-, and monosaccharides and polyols with 76 patients at baseline, 68 patients at week 4 and 47 at month 6. Diary habits were registered digitally [30]. Data normally distributed were presented as mean ±standard deviation (SD) and calculated by Independent-Sample or Paired-Samples T Test*. Data not normally distributed were given as median and interquartile and calculated by Wilcoxon Signed Ranks for comparisons within the groups and Mann-Whitney U test* for comparison between baseline and the differences of the two groups. $P \leq 0.05$ was considered statistically significant.

Supplementary Table S5. Gastrointestinal symptoms before, during and after the dietary intervention

	**SSRD N = 77**			**Low FODMAP N = 78**			**P-value***
VAS-IBS (mm)	**Value**	**FDR-value**	**Difference**	**Value**	**FDR-value**	**Difference**	
**Abdominal pain** 5 (1–13)							
Baseline	47(28-64)	-	-	50(32-65)	-	-	0.368
2 weeks	17(8-30)	0.0015	-27(-47-(-9))	22(13-40)	<0.001	-19(-36-(-3))	0.252
4 weeks	16(0-31)	0.0015	-24(-44-(-9))	13(0-27)	<0.001	-30(-53-(-8))	0.425
6 months	32(19-63)	0.003	-6(-28-3)	30(16-54)	<0.001	-16(-38-4)	0.270
**Diarrhea** 3 (0–10)							
Baseline	53(19-73)	-	-	37(4-74)	-	-	0.245
2 weeks	15(4-48)	<0.001	-14(-51-0)	10(0-37)	0.0015	-14(-38-0)	0.793
4 weeks	17(3-39)	<0.001	-8(-48-2)	8(0-24)	0.0015	-16(-53-0)	0.633
6 months	31(8-68)	<0.001	-12(-39-0)	11(3-44)	0.008	-8(-30-7)	0.457
**Constipation** 6 (2–16)							
Baseline	53(6-72)	-	-	54(10-76)	-	-	0.439
2 weeks	16(2-50)	0.0015	-12(-36-2)	20(0-68)	0.022	-6(-28-4)	0.510
4 weeks	16(2-43)	0.0015	-8(-46-0)	21(0-55)	0.003	-13(-33-0)	0.815
6 months	22(0-61)	0.121	-3(-24-12)	42(2-70)	0.022	-4(-32-3)	0.528
**Bloating and flatulence** 10 (2–23)							
Baseline	73(58-88)	-	-	73(54-86)	-	-	0.677
2 weeks	34(18-53)	0.0015	-37(-53-(-9))	23(13-50)	<0.001	-39(-56-(-16))	0.469
4 weeks	24(10-54)	0.0015	-43(-63-(-11)	19(8-50)	<0.001	-44(-61-(-25))	0.359
6 months	62(30-75)	0.002	-15(-38-14)	56(33-70)	<0.001	-18(-33-(-3))	0.416
**Vomiting and nausea** 2 (0–4)							
Baseline	13(2-34)	-	-	13(1-36)	-	-	0.957
2 weeks	6(0-12)	0.0015	-7(-20-0)	4(0-12)	0.0015	-7(-22-0)	0.773
4 weeks	3(0-12)	0.0015	-6(-15-0)	0(0-11)	0.0015	-7(-21-0)	0.743
6 months	8(1-21)	0.002	-4(-14-1)	5(0-21)	0.009	-2(-17-1)	0.892
**Intestinal symptom´s influence on daily life** 2 (0–14)							
Baseline	74(57-84)	-	-	70(54-84)	-	-	0.694
2 weeks	29(15-60)	<0.001	-36(-52-(-11))	30(17-60)	<0.001	-28(-50-(-15))	0.688
4 weeks	24(12-62)	<0.001	-30(-60-(-10))	22(10-50)	<0.001	-33(-53-(-18))	0.593
6 months	40(23-76)	<0.001	-12(-45-0)	48(24-68)	<0.001	-26(-43-(-2))	0.492
**Psychological well-being** 5 (2–15)							
Baseline	39(15-65)	-	-	45(16-59)	-	-	0.708
2 weeks	24(11-42)	0.0015	-7(-26-0)	27(8-46)	0.0315	-4(-25-5)	0.339
4 weeks	20(5-32)	0.0015	-12(-29-(-2)	18(2-34)	0.003	-13(-32-1)	0.788
6 months	22(11-50)	0.009	-6(-20-4)	26(8-39)	0.043	-2(-30-7)	0.911
IBS-SSS							
**Total IBS-SSS**							
Baseline	301(233-348)	-	-	300(238-360)	-	-	0.845
2 weeks	136(87-223)	<0.001	-138(-212-(-82))	151(100-232)	<0.001	-110(-188-(-68))	0.310
4 weeks	119(66-230)	<0.001	-146(-240-(-88))	116(63-176)	<0.001	-153(-231-(-90))	0.585
6 months	204(146-234)	<0.001	-55(-130-4)	220(144-301)	<0.001	-93(-181-(-20))	0.069

SSRD = starch-and sucrose-reduced diet with 72 patients at week 2 and 4 and 53 at month 6. Low FODMAP = low content of fermentable oligo-, di-, and monosaccharides and polyols with 77 patients at baseline, 71 patients at week 2 and 4 and 49 at month 6. Gastrointestinal symptoms estimated by irritable bowel syndrome -severity scoring system (IBS-SSS) [26]. and visual analog scale for irritable bowel syndrome (VAS-IBS) [27]. Reference values for healthy within brackets [32]. Values are given as median and interquartile. Wilcoxon Signed Ranks for comparisons within the groups and Mann-Whitney U test* for comparison between baseline and the differences of the two groups. P-values adjusted for false discovery rate (FDR) set at 5% according to the Benjamini-Hochberg method [33] were performed for the calculations within the group. $P \leq 0.05$ was considered statistically significant.

Supplementary Table S6. The effect of dietary intervention at week 4 depending on IBS subgroup

	Δ Mean ± SD	n	ANOVA with Bonferroni for IBS subgroups/FBD		
			**IBS-C**	**IBS-M**	**FBD**
SSRD					
**Diarrhea**					
IBS-D	-42±39	27	0.010	0.022	0.103
IBS-C	-7±19	13		1.000	1.000
IBS-M	-14±31	21			1.000
FBD	-12±20	9			
**Constipation**					
IBS-D	-4±28	27	0.478	0.016	0.233
IBS-C	-22±38	13		1.000	1.000
IBS-M	-31±29	21			1.000
FBD	-28±32	9			
Low FODMAP					
**Diarrhea**					
IBS-D	-39±38	15	0.009	1.000	0.139
IBS-C	-1±12	11		0.020	1.000
IBS-M	-32±30	28			0.339
FBD	-13±23	12			
**Constipation**					
IBS-D	-5±13	15	0.778	0.704	1.000
IBS-C	-22±29	12		1.000	1.000
IBS-M	-19±29	28			1.000
FBD	-13±35	12			

SSRD = starch-and sucrose-reduced diet with 77 patients at baseline, 72 at week 4, and 53 at month 6. Low FODMAP = low content of fermentable oligo-, di-, and monosaccharides and polyols with 78 patients at baseline, 72 at week 4, and 49 at month 6. IBS-D = diarrhea-predominated irritable bowel syndrome (IBS), IBS-M = mixed IBS, IBS-C = constipation -predominated IBS, FBD = unspecific functional bowel disorder. Values are given as mean ± standard deviation (SD) and numbers (n). $P \leq 0.05$ was considered statistically significant.

Supplementary table S7. Extraintestinal symptoms before, during and after the dietary intervention

	**SSRD N = 77**			**Low FODMAP N = 78**			**P-value***
Extraintestinal IBS-SSS	**Value**	**FDR-value**	**Difference**	**Value**	**FDR-value**	**Difference**	
**Difficulties to eat a whole meal**							
Baseline	10(2-26)	-	-	6(0-22)	-	-	0.267
2 weeks	4(0-12)	0.003	-3(-13-3)	3(0-13)	0.003	-2(-11-0)	0.951
4 weeks	2(0-13)	0.003	-4(-16-1)	0(0-9)	0.0075	-3(-12-0)	0.629
6 months	4(0-14)	0.074	-2(-13-4)	2(0-18)	0.053	-1(-10-0)	0.940
**Headache**							
Baseline	33(10-66)	-	-	27(9-58)	-	-	0.737
2 weeks	14(5-36)	0.0015	-5(-30-2)	15(2-47)	<0.001	-6(-22-0)	0.993
4 weeks	14(2-32)	0.0015	-9(-27-2)	12(0-35)	<0.001	-9(-31-0)	0.855
6 months	24(10-55)	0.185	-1(-21-8)	20(4-50)	0.001	-4(-21-2)	0.324
**Back pain**							
Baseline	20(4-50)	-	-	28(4-65)	-	-	0.395
2 weeks	6(0-29)	0.0015	-6(-21-0)	14(0-39)	0.0015	-2(-26-2)	0.409
4 weeks	6(0-30)	0.0015	-7(-22-0)	4(0-35)	0.0015	-7(-32-0)	0.998
6 months	23(4-58)	0.157	-4(-18-8)	24(4-70)	0.675	0(-14-12)	0.501
**Fatigue**							
Baseline	57(30-81)	-	-	74(48-84)	-	-	0.055
2 weeks	33(16-68)	0.0015	-14(-27-0)	47(19-70)	<0.001	-12(-32-0)	0.660
4 weeks	27(9-56)	0.0015	-18 (-32-(-2))	37(14-60)	<0.001	-19(-38-(-3))	0.712
6 months	49(18-68)	0.004	-7(-20-4)	48(19-69)	<0.001	-13(-27-0)	0.128
**Belching/excess wind**							
Baseline	72(48-85)	-	-	75(52-87)	-	-	0.621
2 weeks	24(10-66)	<0.001	-21(-51-(-6))	37(14-66)	<0.001	-23(-44-(-6))	0.804
4 weeks	14(6-40)	<0.001	-41(-67-(-6))	21(8-45)	<0.001	-41(-59-(-19))	0.878
6 months	47(20-68)	<0.001	-13(-33-(-2))	48(22-70)	<0.001	-15(-37-(-2))	0.599
**Reflux**							
Baseline	20(7-50)	-	-	20(2-60)	-	-	0.678
2 weeks	5(0-18)	0.0015	-12(-27-0)	7(0-35)	0.0015	-6(-30-0)	0.274
4 weeks	4(0-20)	0.0015	-12(-28-0)	3(0-26)	0.0015	-10(-30-0)	0.945
6 months	11(4-26)	0.002	-6(-20-2)	21(2-54)	0.013	-3(-18-2)	0.727
**Urinary urgency**							
Baseline	14(2-64)	-	-	22(4-64)	-	-	0.491
2 weeks	7(0-24)	0.0015	-7(-24-0)	7(0-33)	0.0015	-8(-29-0)	0.598
4 weeks	4(0-23)	0.0015	-6(-34-0)	3(0-22)	0.0015	-13(-44-0)	0.290
6 months	18(0-44)	0.003	-7(-22-1)	16(0-53)	0.003	-7(-19-0)	0.975
**Leg pain**							
Baseline	2(0-9)	-	-	0(0-18)	-	-	0.995
2 weeks	1(0-15)	0.422	0(-4-2)	0(0-10)	0.015	0(-3-0)	0.776
4 weeks	0(0-7)	0.072	0(-4-0)	0(0-5)	0.054	0(-4-0)	0.564
6 months	2(0-12)	0.774	0(-2-2)	0(0-14)	0.573	0(-2-2)	0.682
**Muscle/joint pain**							
Baseline	25(5-56)	-	-	30(4-72)	-	-	0.506
2 weeks	11(0-46)	0.003	-5(-20-1)	18(0-57)	0.021	-1(-20-4)	0.616
4 weeks	13(0-30)	0.003	-10(-27-0)	12(0-39)	0.003	-3(-35-1)	0.699
6 months	23(5-53)	0.032	-3(-17-4)	19(4-70)	0.084	-2(-18-7)	0.755
**Total extraintestinal IBS-SSS**							

Baseline	160(110-208)	-	-	172(120-242)	-	-	0.268
2 weeks	96(50-154)	<0.001	-60(-89-(-20))	115(55-156)	<0.001	-54(-82-(-30))	0.852
4 weeks	91(28-140)	<0.001	-72(-112-(-41))	77(44-136)	<0.001	-83(-118-(-44))	0.408
6 months	127(71-191)	<0.001	-44(-75-2)	133(78-214)	<0.001	-36(-59-(-10))	0.977

SSRD = starch-and sucrose-reduced diet with 72 patients at week 2 and 4 and 53 at month 6. Low FODMAP = low content of fermentable oligo-, di-, and monosaccharides and polyols with 77 patients at baseline, 71 patients at week 2 and 4 and 49 at month 6. Symptoms estimated by irritable bowel syndrome -severity scoring system (IBS-SSS) [26]. Values are given as median and interquartile. Wilcoxon Signed Ranks for comparisons within the groups and Mann-Whitney U test* for comparison between baseline and the differences of the two groups. P-values adjusted for false discovery rate (FDR) set at 5% according to the Benjamini-Hochberg method [33] were performed for the calculations within the group. $P \leq 0.05$ was considered statistically significant.

Supplementary Table S8. Correlations between improvements of gastrointestinal and extraintestinal symptoms

	**Diff_Total IBS_SSS_2 weeks**	**Diff_Total IBS_SSS_4 weeks**	**Diff_Total IBS_SSS_6 months**
**Diff_Total Extraintestinal symptoms**	r=0.390 p<0.001 FDR<0.001	r=0.409 p<0.001 FDR<0.001	r=0.439 p<0.001 FDR<0.001
Diff_vomiting	r=0.151 p=0.073 FDR=0.073	r=0.275 p<0.001 FDR=0.003	r=0.262 p=0.008 FDR=0.012
Diff_difficulties to eat a whole meal	r=0.252 p=0.003 FDR=0.009	r=0.184 p=0.028 FDR=0.028	r=0.234 p=0.018 FDR=0.027
Diff headache	r=0.177 p=0.036 FDR=0.054	r=0.224 p=0.011 FDR=0.033	r=0.164 p=0.100 FDR=1.00
Diff_back pain	r=0.094 p=0.268	r=0.094 p=0.266	r=0.043 p=0.669
Diff_fatigue	r=0.363 p<0.001 FDR=0.0015	r=0.431 p<0.001 FDR=0.0015	r=0.204 p=0.040 FDR=0.040
Diff_belching/excess wind	r=0.239 p=0.004 FDR=0.004	r=0.419 p<0.001 FDR=0.0015	r=0.324 p<0.001 FDR=0.0015
Diff_reflux	r=0.225 p=0.007 FDR=0.021	r=0.137 p=0.102 FDR=0.102	r=0.234 p=0.018 FDR=0.027
Diff_urinary urgency	r=0.108 p=0.204 FDR=0.204	r=0.198 p=0.018 FDR=0.054	r=0.184 p=0.064 FDR=0.096
Diff_leg pain	r=0.025 p=0.765	r=0.091 p=0.279	r=0.023 p=0.817
Diff_muscle/joint pain	r=-0.012 p=0.890 FDR=0.890	r=0.175 p=0.037 FDR=0.111	r=0.168 p=0.091 FDR=0.1365
Diff_psychological well-being	r=0.272 p=0.001 FDR=0.001	r=0.309 p<0.001 FDR<0.001	r=0.366 p<0.001 FDR<0.001
Diff_weight	-	r=0.041 p=0.625 FDR=0.625	r=0.257 p=0.010 FDR=0.020
Diff_BMI	-	r=0.022 p=0.792 FDR=0.792	r=0.247 p=0.013 FDR=0.026
Diff_waist_circumference	-	r=-0.086 p=0.312	r=0.021 p=0.838
Diff_systolic_blood_pressure	-	r=0.115 p=0.171	r=0.172 p=0.086
Diff_diastolic_blood_pressure	-	r=0.025 p=0.769	r=0.176 p=0.079
Diff_sugar_cravings	-	r=-0.070 p=0.408	r=0.119 p=0.237

Correlations between decreases in specific and total extraintestinal symptoms and decreases of total gastrointestinal symptoms (total IBS-SSS) at week 2, week 4, and month 6 [26]. 155 patients at baseline (154 for symptoms), 144 patients at week 4 for BMI, waist circumference, and blood pressure and 143 for symptoms, and 102 patients at month 6. Spearman´s correlation test performed in the whole cohort. Variables with significant p-values were adjusted for false discovery rate (FDR) set at 5% according to the Benjamini-Hochberg method [33]. P≤0.05 was considered statistically significant.

Supplementary Table S9. Anthropometric data

	SSRD<br>N = 77			Low FODMAP<br>N = 78			P-value*
**Variables**	**Value**	**FDR-value**	**Difference**	**Value**	**FDR-value**	**Difference**	
**Weight** (kg)							
Baseline	71.5(63.6-82.8)	-	-	68.6(63-83.4)	-	-	0.513
4 weeks	70(63.2-81)	0.002	-1.6(-2.4-(-0.4))	67.8(62.5-82.7)	0.002	-0.8(-1.6-(-0.1))	0.006
6 months	74.1(66.6-85.7)	0.516	-0.2(-1.4-1.2)	68.6(62.8-80.8)	0.079	-0.3(-1.6-0.6)	0.438
**BMI** (kg/m^2)							
Baseline	25.14(22.64-28.45)	-	-	24.68(22.13-27.64)	-	-	0.538
4 weeks	24.8(21.97-27.6)	0.002	-0.55(-0.86-(-0.15))	24.63(22-27.32)	0.002	-0.26(-0.56-(-0.03))	0.005
6 months	25.95(22.66-28.57)	0.504	-0.07(-0.53-0.44)	25.08(22.05-26.76)	0.089	-0.11(-0.54-0.23)	0.526
**Waist circumference** (cm)							
Baseline	88(76-97)	-	-	86(79-94.8)	-	-	0.831
4 weeks	86(75-94)	0.002	-2(-4-0)	85(79-93)	0.002	-2(-3-1)	0.981
6 months	89(77.5-97)	0.022	-1(-4-1)	85.5(80-93.5)	0.038	-1(-3-1)	0.758
**Systolic Blood Pressure** (mmHg)							
Baseline	125(114-139)	-		126(116-139)	-		0.657
4 weeks	123(114-135)	0.194	-2(-10-6)	124(113-135)	0.048	-3(-8-3)	0.762
6 months	127(116-138)	0.588	-1(-7-6)	126(117-136)	0.138	-3(-12-8)	0.399
**Diastolic Blood Pressure** (mmHg)							
Baseline	81(72-88)	-		81(74-90)	-		0.403
4 weeks	78(70-84)	0.012	-3(-6-3)	80(73-85)	0.002	-4(-8-2)	0.225
6 months	80(76-86)	0.575	-2(-6-5)	80(72-86)	0.044	-1(-10-3)	0.190
**Sugar craving** (mm)							
Baseline	66(40-85)	-	-	60(29-80)	-	-	0.384
4 weeks	34(17-67)	0.002	-15(-41-0)	41(22-69)	0.002	-8(-23-5)	0.050
6 months	53(31-72)	0.058	-7(-23-10)	48(28-72)	0.246	-2(-10-8)	0.448
**Saturation** (mm)							
Baseline	74(52-93)	-	-	77(68-86)	-	-	0.833
4 weeks	83(69-93)	0.214	4(-12-24)	80(62-90)	0.688	1(-10-12)	0.261
6 months	77(69-90)	0.473	0(-12-16)	72(62-87)	0.688	-2(-13-16)	0.275

BMI = body mass index. SSRD = starch-and sucrose-reduced diet with 77 patients at baseline, 72 at week 4, and 53 at month 6. Low FODMAP = low content of fermentable oligo-, di-, and monosaccharides and polyols 72 patients at week 2 and 4 (except sugar craving and saturation with 77 at baseline and 71 at week 2 and 4) and 49 at month 6. Sugar craving and saturation estimated on visual analog scales of 100 mm, with higher scores meaning most craving and saturation. Values are given as median and interquartile. Wilcoxon Signed Ranks for comparisons within the groups and Mann-Whitney U test* for comparison between baseline and the differences of the two groups. P-values adjusted for false discovery rate (FDR) set at 5% according to the Benjamini-Hochberg method [33] were performed for the calculations within the group. $P \leq 0.05$ was considered statistically significant.

Supplementary Figure S1

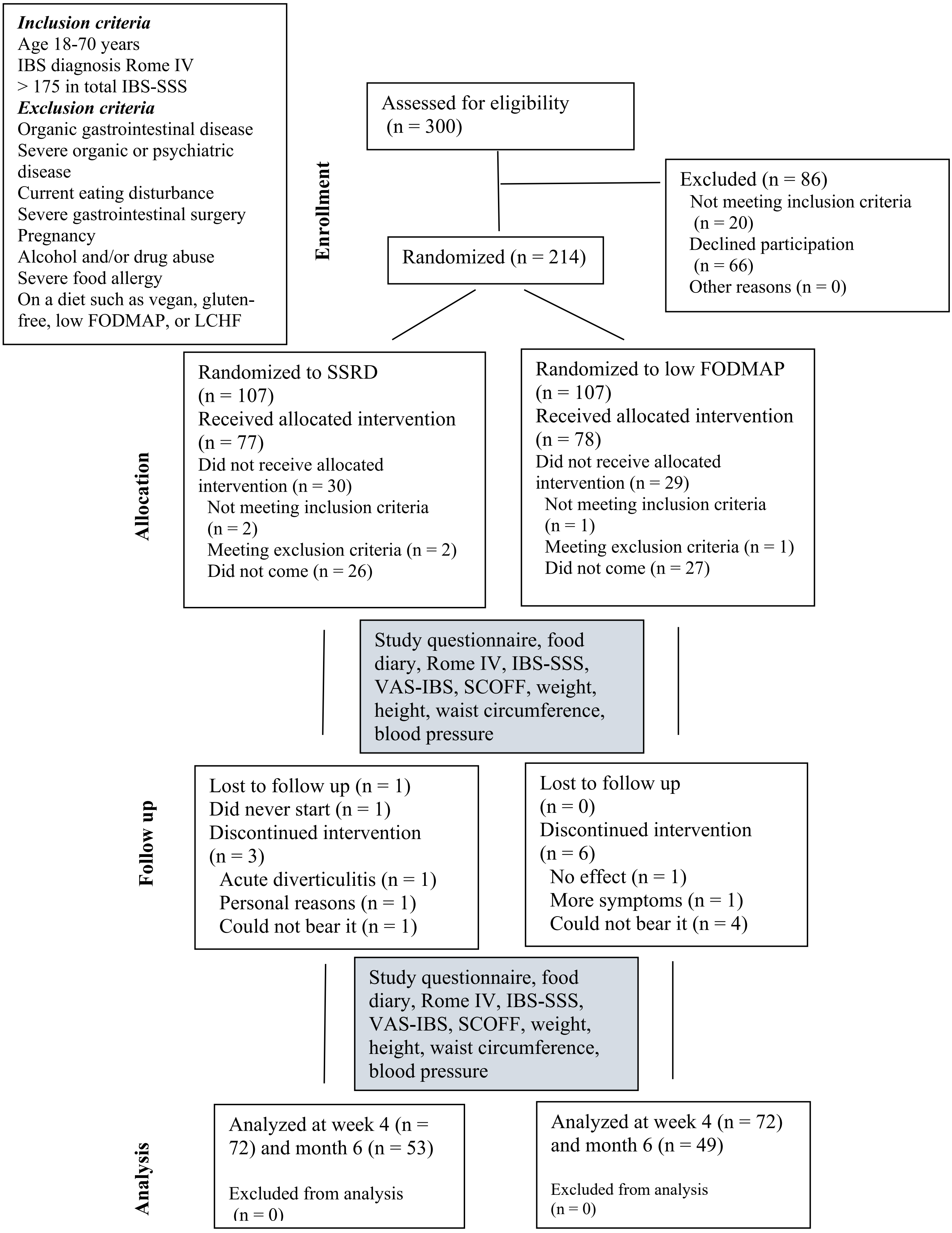

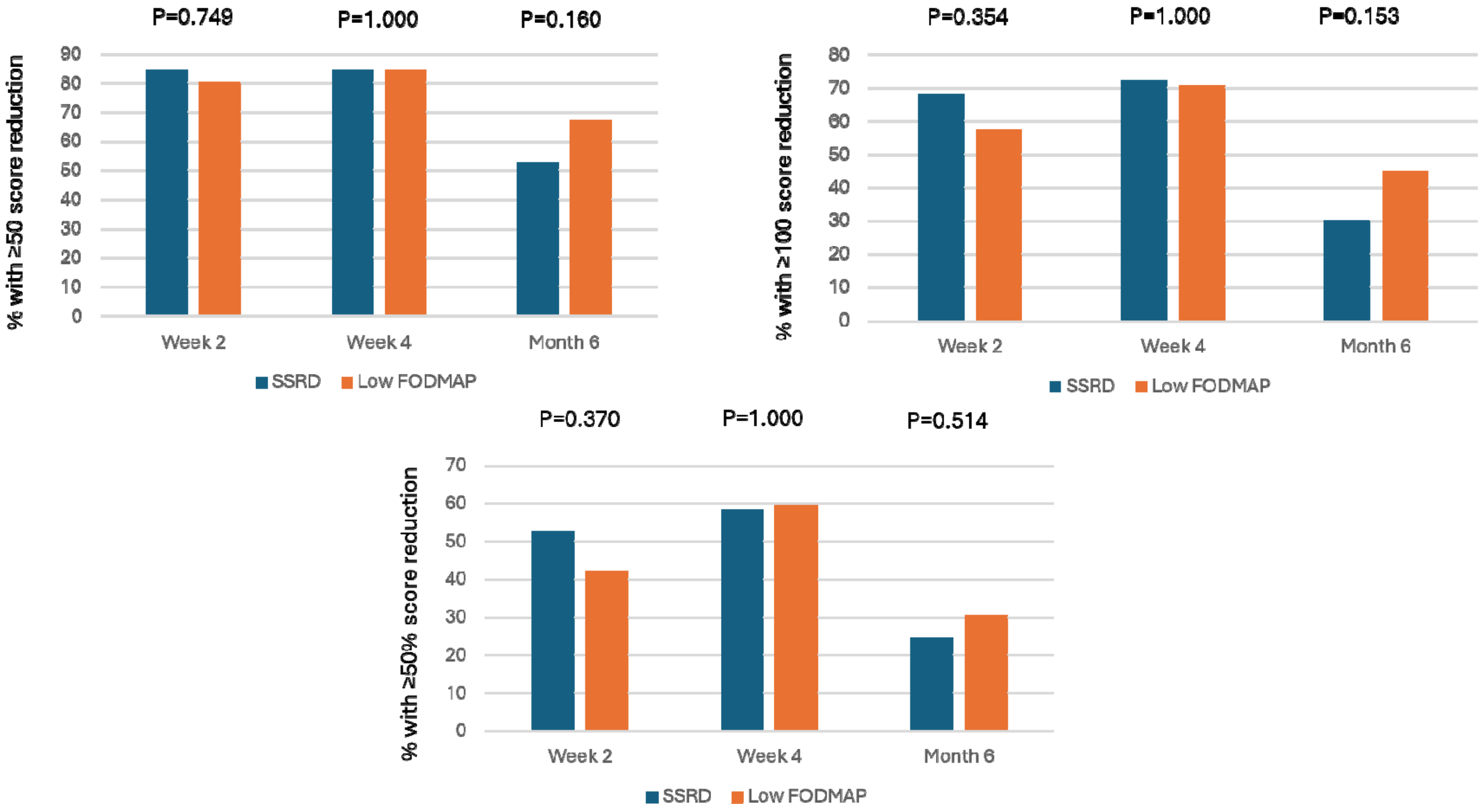

Supplementary Figure S2
